# No Impairment in Host Defense against *Streptococcus pneumoniae* in Obese CPE*^fat/fat^* Mice

**DOI:** 10.1371/journal.pone.0106420

**Published:** 2014-09-09

**Authors:** Peter Mancuso, Edmund O′Brien, Joseph Prano, Deepti Goel, David M. Aronoff

**Affiliations:** 1 Department of Environmental Health Sciences, University of Michigan, Ann Arbor, Michigan, United States of America; 2 Graduate Program in Immunology, University of Michigan, Ann Arbor, Michigan, United States of America; 3 Division of Infectious Diseases, Department of Medicine, Vanderbilt University, Nashville, Tennessee, United States of America; Hannover School of Medicine, Germany

## Abstract

In the US and globally, dramatic increases in the prevalence of adult and childhood obesity have been reported during the last 30 years. In addition to cardiovascular disease, type II diabetes, and liver disease, obesity has recently been recognized as an important risk factor for influenza pneumonia. During the influenza pandemic of 2009, obese individuals experienced a greater severity of illness from the H1N1 virus. In addition, obese mice have also been shown to exhibit increased lethality and aberrant pulmonary inflammatory responses following influenza infection. In contrast to influenza, the impact of obesity on bacterial pneumonia in human patients is controversial. In this report, we compared the responses of lean WT and obese CPE*^fat/fat^* mice following an intratracheal infection with *Streptococcus pneumoniae*, the leading cause of community-acquired pneumonia. At 16 weeks of age, CPE*^fat/fat^* mice develop severe obesity, hyperglycemia, elevated serum triglycerides and leptin, and increased blood neutrophil counts. There were no differences between lean WT and obese CPE*^fat/fat^* mice in survival or lung and spleen bacterial burdens following intratracheal infection with *S. pneumoniae*. Besides a modest increase in TNF-α levels and increased peripheral blood neutrophil counts in CPE*^fat/fat^* mice, there were not differences in lung or serum cytokines after infection. These results suggest that obesity, accompanied by hyperglycemia and modestly elevated triglycerides, at least in the case of CPE*^fat/fat^* mice, does not impair innate immunity against pneumococcal pneumonia.

## Introduction

The prevalence of obesity has increased dramatically during the last three decades with 35 percent of the US adult population having a body mass index (BMI) of 30 kg/m^2^ or greater [1]. While obesity is recognized as a significant risk factor for type II diabetes, hypertension, and cardiovascular disease, it is also a significant contributing factor to the pathogenesis of pulmonary diseases such as asthma, obstructive sleep apnea, chronic obstructive pulmonary disease (chronic bronchitis), and a greater severity of illness and death due to influenza H1N1 [2]–[6].

The death toll from the H1N1 pandemic of 2009 has been estimated to be more than 284,000 worldwide [7]. Although the majority of cases were relatively mild and self-limiting, the severity of illness and mortality were greater among the 30–50 age group [8]. In particular, the obese were disproportionately represented among influenza-associated hospitalizations and deaths [9]. This association between obesity and severity of illness and death from H1N1 influenza has been confirmed by many other reports [10]–[14]. Furthermore, it appears that obesity is a risk factor for severity of illness from other strains of influenza and viral pathogens known to infect the respiratory tract [15], [16]. Importantly, the ability of obesity to diminish host defense against influenza infections has been confirmed in robust and carefully controlled studies using obese mice challenged with the pandemic H1N1 and H3N2 strains of the influenza virus [15], [17], [18]. Whether or not obesity is associated with a greater severity of illness with bacterial pneumonia is less certain [6], [19].

Previously, we reported that obese leptin-deficient *ob/ob* mice exhibited greater mortality following an intratracheal challenge with either *K. pneumoniae* or *S.pneumoniae* [20], [21]. In these studies, greater mortality in the *ob/ob* mouse was associated with impaired pulmonary bacterial clearance and attenuated alveolar macrophage and neutrophil phagocytosis and killing of bacteria, and the elaboration of reactive oxygen intermediates [22]. In addition, many other reports have demonstrated that *ob/ob* mice exhibit host defense defects in response to several other bacterial, mycobacterial, amoeba, and fungal infections [23]–[28]. However, leptin deficiency disables host defense, in the absence of obesity, and has been demonstrated to restore antimicrobial functions in the presence of obesity in *ob/ob* mice [21], [29]. The effect of obesity on host defense against community-acquired pneumonia in humans is controversial and appropriate animal models have not been used to address this important question [19]. In the current study, we compared the responses of lean wild type (WT) and obese CPE*^fat/fat^* mice, which lack a functional carboxypeptidase enzyme, following an intratracheal infection with *Streptococcus pneumoniae*, the most common cause of community-acquired pneumonia [30].

## Materials and Methods

### Ethics statement

All animals were treated according to National Institutes of Health guidelines for the use of experimental animals with the approval of the University of Michigan Committee for the Use and Care of Animals (Protocol Number: #PRO00003932).

### Animals

Female CPE*^fat/fat^* mice, bred on a C57BL/6 background and age-matched, female C57BL/6 wild type (WT) animals, were purchased from The Jackson Laboratory, Bar Harbor, ME. All mice were 16–18 weeks of age prior to their use in all the experiments performed for these studies. All mice were maintained in the University of Michigan Unit for Laboratory Animal Medicine, maintained on Formulab 5008 rodent chow (LabDiet, Brentwood, MO).

### Murine model of pneumococcal pneumonia

*S. pneumoniae* serotype 3, 6303 (American Type Culture Collection, Manassas, VA) was grown to mid-log phase in Todd-Hewett broth, washed in PBS, and serially diluted in sterile saline. Following anesthesia with ketamine (80 mg/kg) and xylazine (10 mg/kg) delivered via an intraperitoneal injection, a midline incision was made to expose the trachea, a 30-µl inoculum containing 50,000 CFU *S*. ***pneumoniae*** was administered via the trachea using a 26-gauge needle, and the wound was closed using surgical glue (Vetbond, 3 M, St. Paul, MN) [31]. Following infection, mice were warmed by placing their cage on a heating pad and closely observed every 10 min until they recovered from the anesthesia. For the duration of the lethality study, they were observed for survival every 4 hours during the day. Moribund animals (i.e. staggered gate, ruffled fur, unable to reach water or food) were euthanized by CO2 asphyxiation to ameliorate suffering. In a separate group of mice, bacterial growth in lung and spleen homogenates was determined 24 and 48 h after infection using serial dilutions plated on blood agar as previously described [29].

### Lung bronchoalveolar lavage fluid (BALF) and blood cytokine determinations

BALF and blood obtained from mice 24 and 48 h after pneumococcal infection were evaluated for GM-CSF(blood only), IL-1β, IL-6, IL-10, IL-12 p70, MIP-2, MCP-1, and TNF-α by ELISA (R&D Duoset, R&D Systems) performed by the University of Michigan Cancer Center Cellular Immunology Core as previously described [32]. Leptin levels (blood only) were determined according to the manufacturer's instructions using a commercially available ELISA kit (Millipore, St. Charles, MO).

### Blood glucose, serum triglycerides, and leukocyte determinations

Blood was obtained from mice by cardiac puncture following euthanasia by an overdose of CO_2_ for glucose measurements and leukocyte counts. Blood glucose was assessed at baseline using a glucometer (Glucometer Elite; Bayer, Elkhart, IN). Serum triglyceride levels were measured using the GPO kit from Raichem (Clinica Corp., San Marcos, CA), with glycerol as a standard according to the manufacturer's instructions. Leukocyte counts were performed after red blood cell lysis (Unopette Microcollection System; Becton-Dickson, Rutherford, NJ) and a Hemavet cell analyzer (Drew Scientific) operated by the University of Michigan Unit for Laboratory Animal Medicine Animal Diagnostic Laboratory.

### Statistical analyses

Statistical analyses were conducted using Prism 6.0 software (GraphPad Software, La Jolla, CA). Survival differences were assessed using the Mantel-Cox log-rank test. Where appropriate, mean values were compared using a Student *t*-test. Differences were considered significant if *P*≤0.05. All experiments were performed at least three separate times unless otherwise noted in the figure legend. Data are presented as mean values ± standard error of the mean unless noted otherwise.

## Results

### Obesity and metabolic abnormalities in CPE*^fat/fat^* mice

**Figure 1:**
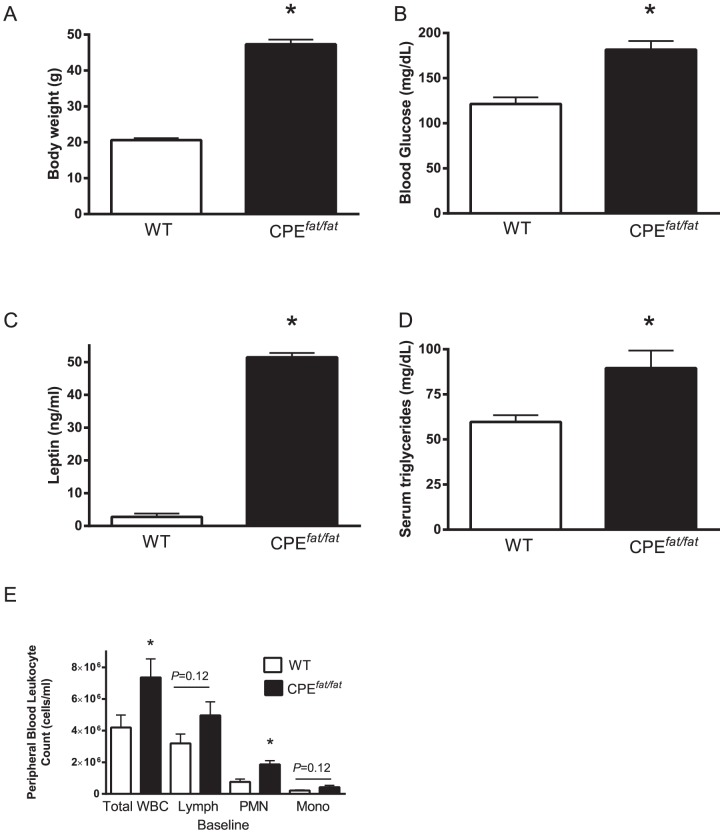
Body weight (A), blood glucose (B), serum leptin (C), triglycerides (D), and white blood cell counts (E) in 16-wk-old female wild type (WT) and carboxypeptidase E *^fat/fat^* (CPE*^fat/fat^*) mice at baseline. *,<0.05 vs WT using student's *t*-test. N = 4 mice per group (A-D) and N = 10 mice per group (E).

In CPE*^fat/fat^* mice, the obese phenotype arises from the lack of carboxypeptidase E, an enzyme that plays an essential role in processing prohormones and proneuropeptides known to regulate appetite and energy expenditure [33]–[35]. As a consequence of the absence of carboxypeptidase, CPE*^fat/fat^* mice exhibit hyperphasia, reduced locomotor activity, and reduced energy expenditure compared with wild type animals [34]. As shown in Figure 1, the body weights of CPE*^fat/fat^* mice were 2-fold greater than that of WT animals and the increased body mass has previously been attributed to greater fat mass [34]. In addition, the CPE*^fat/fat^* mice were hyperglycemic at 16 wks of age and had substantially higher levels of leptin and serum (Figure 1B, C and D). Interestingly, we also observed greater total white blood cell (WBC) and neutrophil (PMN) counts in CPE*^fat/fat^* mice (Figure 1E). In total, the CPE*^fat/fat^* mouse is obese and hyperglycemic with elevated triglycerides, leptin, and peripheral WBC and PMN counts when maintained on a normal chow diet at 16 wks of age.

### Differences in weight loss but not survival or bacterial burdens in WT and CPE*^fat/fat^* mice following intratracheal *S. pneumoniae* infection

**Figure 2:**
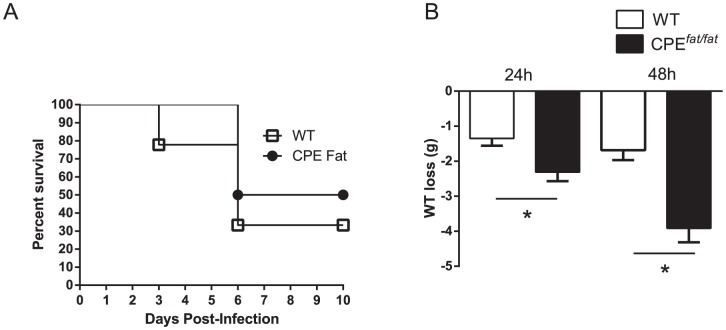
Survival of WT and CPE*^fat/fat^* mice following *S. pneumoniae* infection (A). Body weight loss, expressed as total weight loss, 24 and 48 h post-infection (B). Mice were infected with 5×10^4^ CFUs of *S. pneumonia* via the intratracheal route and monitored for survival for 10 days. N = 6–9 mice per group from 2 independent experiments. Survival was evaluated using the log-rank test. *, *p*<0.05, total weight loss for WT vs CPE*^fat/fat^* mice post-infection using a student's *t*-test, n = 5 (24 h) and 15 (48 h) mice per group.

**Figure 3:**
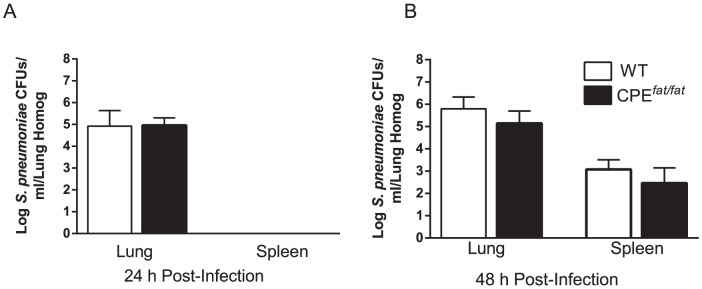
Bacterial burdens in WT and CPE*^fat/fat^* mice 24 (A) and 48 h (B) following *S. pneumoniae* challenge. Mice were infected with 5×10^4^ CFUs of *S. pneumoniae* via the intratracheal route and bacterial loads in tissue homogenates were determined by enumerating CFUs as described in the *Materials and Methods* section. Statistical comparisons were performed using the students *t*-test. n = 5 (24 h) and 15 (48 h) mice per group.

Since the risk of community-acquired pneumonia among obese individuals in clinical and epidemiologic studies is uncertain [6], we assessed survival and weight loss in WT and CPE*^fat/fat^* mice following *S. pneumonia* challenge. As shown in Figure 2, the differences between WT and CPE*^fat/fat^* mice in survival were modest and did not reach statistical significance (p = 0.37). However, we did find greater absolute weight loss after infection in CPE*^fat/fat^* mice at both 24 and 48 h post-infection (Figure 2B). In contrast, we did not find differences in % weight loss (from baseline) post-infection (data not shown). Since differences in survival may not reflect potential differences in host defense, we also examined lung and spleen bacterial burdens after infection. As shown in Figure 3, pulmonary and spleen bacterial loads were not different between WT and CPE*^fat/fat^* mice at 24 or 48 h after *S. pneumoniae* challenge. Based on these results, CPE*^fat/fat^* did not exhibit impairments in survival or pulmonary clearance of *S. pneumoniae.*

### Effect of *S. pneumonia*e challenge on cytokines in BALF

**Figure 4:**
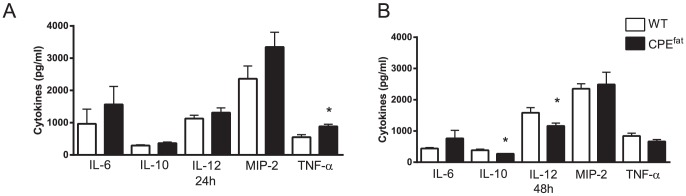
Cytokines in bronchoalveolar lavage fluid (BALF) from WT and CPE*^fat/fat^* mice 24 (A) and 48 h (B) post-infection. Mice were challenged with 5×10^4^ CFUs of *S. pneumoniae* via the intratracheal route and BALF was obtained 24 and 48 h post- infection and cytokine levels were determined as described in the *Materials and Methods* section. n = 5–7 mice per group. *, *p<0.05* vs WT using a student's *t*-test.

Excess adipose tissue has been shown to produce cytokines that significantly contribute to a chronic state of low-grade systemic inflammation in obese humans and animals [36]. Whether or not pulmonary cytokine production is differentially regulated in CPE*^fat/fat^* mice during pneumococcal pneumonia has not been evaluated. Cytokine levels at baseline in BALF were below the limit of detection (data not shown). As shown in Figure 4, higher levels of TNF-α were observed in the BALF of CPE*^fat/fat^* mice with modestly elevated levels of IL-6, IL-10, IL-12, and MIP-2 that did not reach statistical significance 24 h after infection. In contrast, IL-10 and IL-12 levels were lower in CPE*^fat/fat^* mice 48 h post-infection. As was observed at 24 h, IL-6 was elevated in CPE*^fat/fat^* mice 48 h post-infection but this difference was not statistically significant. In total, there were modest differences between WT and CPE*^fat/fat^* mice in pulmonary cytokines following *S. pneumoniae* challenge.

### Impact of obesity on the systemic inflammatory response following *S. pneumoniae* challenge

**Figure 5:**
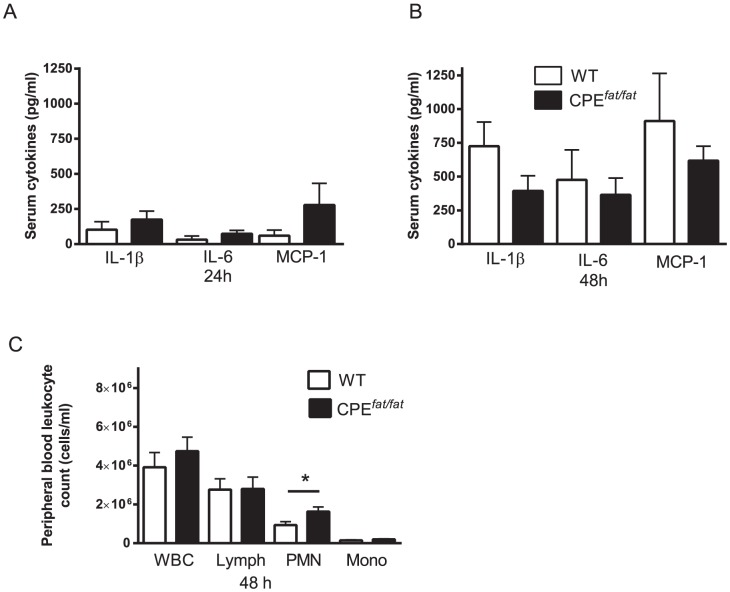
Serum cytokines (A and B) and peripheral blood cell counts (C) in WT and CPE*^fat/fat^* mice following *S. pneumoniae* challenge. WT and CPE*^fat/fat^* mice were challenged with 10^5^ CFUs of *S. pneumoniae* via the intratracheal route and serum was prepared from blood samples obtained 24 and 48 h post-infection as mentioned in the *Materials and Methods* section. n = 5–7 mice per group *, *p*<0.05 vs WT using a student's *t*-test.

Since the elevated levels of systemic proinflammatory cytokines (MCP-1, IL-1β, and IL-6) and increased peripheral blood leukocyte counts have been reported in obese humans and CPE*^fat/fat^* mice, we assessed these cytokines in serum and peripheral blood leukocyte counts after infection [35], [37]. Although there was a trend for elevated levels of IL-1β, IL-6, and MCP-1 in CPE*^fat/fat^* mice 24 h post infection, none of these differences reached statistically significance (Figure 5A). In addition, we did not observe differences in blood GM-CSF (data not shown). In contrast, 48 h after infection, we observed a non-significant trend for higher levels of all cytokines in WT animals which was consistent with the trend for elevated spleen bacterial counts (non-significant trend) in WT mice at this time point (Figure 5B). PMN counts were elevated in CPE*^fat/fat^* mice 48 h after infection. (Figure 5C). In total, we observed elevated PMN counts in the CPE*^fat/fat^* mice with no differences in peripheral blood cytokines during pneumococcal pneumonia.

## Discussion

Obese humans and mice are known to experience a greater severity of illness and death from influenza pneumonia [8], [15], [17], [18], [38], [39]. In this report, we compared the responses of lean and obese CPE*^fat/fat^* mice following an intratracheal challenge with *S. pneumoniae*. Despite severe obesity, hyperglycemia, elevated peripheral blood neutrophil counts, and modest differences in lung cytokines following infection, there were no differences between CPE*^fat/fat^* mice and WT animals in survival or pulmonary and spleen bacterial burdens following *S. pneumoniae* challenge. These results suggest that obesity in the CPE*^fat/fat^* mouse, even in the presence of hyperglycemia, does not impair host defense against pneumococcal pneumonia.

While we did not observe differences in bacterial burdens in obese CPE*^fat/fat^* and WT mice following *S. pneumoniae* challenge, it is possible that pulmonary host defense may be impaired in other models of obesity. CPE*^fat/fat^* mice become hyperphagic due to the lack of functional hormones that regulate food intake [40]. The greater weight loss in the CPE*^fat/fat^* mice was likely due to a reduction in hyperphagia since pneumonia is well known to decrease appetite [29]. However, the % weight loss from baseline, which was not different, is a better measure of the severity of infection in murine models of bacterial pneumonia. The mean blood glucose level of 16-wk-old CPE*^fat/fat^* mice was approximately 180 mg/dL. Murine diet induced obesity (DIO) is produced by feeding animals a high fat diet (40–60% kcals from fat) for several weeks (usually 10–26 wks) resulting in obesity and diabetes (blood glucose >200 mg/dL) [41], [42]. DIO mice have been shown to exhibit greater renal bacterial burdens in a model of *S. aureus*-induced sepsis and higher oral bacterial counts following *P. gingivalus* infection [43]. Diabetes, a common comorbidity of obesity, is a well-known risk factor for pneumococcal infections, and poor glucose control in diabetes is known to increase the risk of pneumococcal pneumonia [44], [45]. Hyperglycemia in type II diabetes is known to compromise host defense against cutaneous infections by impairing wound healing, antimicrobial peptide (LL-37)(cathelicidin) production, and epithelial cell proliferation following tissue injury [15], [46], [47]. Therefore, the combination of obesity and diabetes may be required to impair host defense during bacterial pneumonia.

Leukocytosis is frequently observed in obese humans and mice [37], [48]–[51]. While having a greater number of peripheral blood leukocytes, such as PMNs known to ingest and killing bacteria, may enhance host defense against bacterial infections, a profound recruitment of these cells to a site of infection may also induce collateral tissue damage that diminishes bacterial clearance [50]. The elevated PMNs in CPE*^fat/fat^* mice at baseline and after infection did not seem to contribute to differences in bacterial burdens in our study.

Excess adipose tissue may contribute to pulmonary inflammation since levels of systemic cytokines (IL-1β, IL-6, MCP-1, and TNF-α) and acute phase proteins (C-reactive protein and serum amyloid A) are elevated in obese humans and mice [52]. Johnston and co-workers reported enhanced pulmonary inflammatory responses in CPE*^fat/fat^* mice following exposure to ozone that was characterized by increased BAL protein, cytokines (IL-6, KC, MCP-1, and MIP-2), and neutrophil recruitment [35]. In addition, peripheral blood neutrophils and MCP-1 were also elevated in the CPE*^fat/fat^* mice in response to ozone. In our studies, we observed modest increases in TNF-α in BALF at 24 h and elevated peripheral blood neutrophils in CPE*^fat/fat^* mice at baseline and 48 h following an intratracheal challenge with *S.pneumoniae*. While there were trends for increased IL-6, IL-1β, and MCP-1 in CPE*^fat/fat^* mice 24 h after infection, these differences did not reach statistical significance. At 48 h post-infection, the lower levels of IL-10 and IL-12 and trend for reduced TNF-α in CPE*^fat/fat^* mice may have been due to the non-significant trend for lower spleen CFUs. In total, the low-grade inflammatory state observed in the CPE*^fat/fat^* mice did not affect pulmonary host defense or substantially enhance pulmonary, or systemic cytokine production following infection.

In summary, obese CPE*^fat/fat^* mice do not exhibit impairments in host defense against *S. pneumoniae*. These results suggest that obesity, accompanied with modest metabolic abnormalities, does not compromise pulmonary innate immunity against pneumococcal pneumonia.

## References

[1] Ogden CL, Carroll MD, Kit BK, Flegal KM (2013) Prevalence of obesity among adults: United States, 2011–2012. NCHS Data Brief: 1–8. 24152742

[2] Cecere LM, Littman AJ, Slatore CG, Udris EM, Bryson CL, et al (2011) Obesity and COPD: associated symptoms, health-related quality of life, and medication use. COPD 8: 275–284. 21809909 10.3109/15412555.2011.586660 PMC3169653

[3] Gidding SS, Nehgme R, Heise C, Muscar C, Linton A, et al (2004) Severe obesity associated with cardiovascular deconditioning, high prevalence of cardiovascular risk factors, diabetes mellitus/hyperinsulinemia, and respiratory compromise. J Pediatr 144: 766–769. 15192624 10.1016/j.jpeds.2004.03.043

[4] Marcus JA, Pothineni A, Marcus CZ, Bisognano JD (2014) The role of obesity and obstructive sleep apnea in the pathogenesis and treatment of resistant hypertension. Curr Hypertens Rep 16: 411. 24346827 10.1007/s11906-013-0411-y

[5] Sideleva O, Black K, Dixon AE (2013) Effects of obesity and weight loss on airway physiology and inflammation in asthma. Pulm Pharmacol Ther 26: 455–458. 22609067 10.1016/j.pupt.2012.05.002 PMC3502699

[6] Mancuso P (2013) Obesity and respiratory infections: does excess adiposity weigh down host defense? Pulm Pharmacol Ther 26: 412–419. 22634305 10.1016/j.pupt.2012.04.006 PMC3439591

[7] Dawood FS, Iuliano AD, Reed C, Meltzer MI, Shay DK, et al (2012) Estimated global mortality associated with the first 12 months of 2009 pandemic influenza A H1N1 virus circulation: a modelling study. Lancet Infect Dis 12: 687–695. 22738893 10.1016/S1473-3099(12)70121-4

[8] Singanayagam A, Wood V, Chalmers JD (2011) Factors associated with severe illness in pandemic 2009 influenza a (H1N1) infection: implications for triage in primary and secondary care. J Infect 63: 243–251. 21839111 10.1016/j.jinf.2011.07.014

[9] LaRussa P (2011) Pandemic Novel 2009 H1N1 Influenza: What Have We Learned? Semin Respir Crit Care Med 32: 393–399. 21858744 10.1055/s-0031-1283279

[10] Centers for Disease Control and Prevention (CDC) (2009) Intensive-care patients with severe novel influenza A (H1N1) virus infection - Michigan, June 2009. MMWR Morb Mortal Wkly Rep 58: 749–752. 19609249

[11] Vaillant L, La Ruche G, Tarantola A, Barboza P (2009) Epidemiology of fatal cases associated with pandemic H1N1 influenza 2009. Euro Surveill 14. 10.2807/ese.14.33.19309-en 19712643

[12] Fuhrman C, Bonmarin I, Bitar D, Cardoso T, Duport N, et al (2011) Adult intensive-care patients with 2009 pandemic influenza A(H1N1) infection. Epidemiol Infect 139: 1202–1209. 20974021 10.1017/S0950268810002414

[13] Fuhrman C, Bonmarin I, Paty AC, Duport N, Chiron E, et al.. (2010) Severe hospitalised 2009 pandemic influenza A(H1N1) cases in France, 1 July-15 November 2009. Euro Surveill 15. 10.2807/ese.15.02.19463-en 20085690

[14] Jain S, Kamimoto L, Bramley AM, Schmitz AM, Benoit SR, et al (2009) Hospitalized Patients with 2009 H1N1 Influenza in the United States, April–June 2009. New England Journal of Medicine 361: 1935–1944. 19815859 10.1056/NEJMoa0906695

[15] O′Brien KB, Vogel P, Duan S, Govorkova EA, Webby RJ, et al (2012) Impaired wound healing predisposes obese mice to severe influenza virus infection. J Infect Dis 205: 252–261. 22147799 10.1093/infdis/jir729 PMC3244366

[16] Akiyama N, Segawa T, Ida H, Mezawa H, Noya M, et al (2011) Bimodal effects of obesity ratio on disease duration of respiratory syncytial virus infection in children. Allergol Int 60: 305–308. 21430434 10.2332/allergolint.10-OA-0252

[17] Easterbrook JD, Dunfee RL, Schwartzman LM, Jagger BW, Sandouk A, et al (2011) Obese mice have increased morbidity and mortality compared to non-obese mice during infection with the 2009 pandemic H1N1 influenza virus. Influenza Other Respir Viruses 5: 418–425. 21668672 10.1111/j.1750-2659.2011.00254.x PMC3175349

[18] Smith AG, Sheridan PA, Harp JB, Beck MA (2007) Diet-induced obese mice have increased mortality and altered immune responses when infected with influenza virus. J Nutr 137: 1236–1243. 17449587 10.1093/jn/137.5.1236

[19] Phung DT, Wang Z, Rutherford S, Huang C, Chu C (2013) Body mass index and risk of pneumonia: a systematic review and meta-analysis. Obesity Reviews 14: 839–857. 23800284 10.1111/obr.12055

[20] Mancuso P, Gottschalk A, Phare SM, Peters-Golden M, Lukacs NW, et al (2002) Leptin-deficient mice exhibit impaired host defense in Gram-negative pneumonia. J Immunol 168: 4018–4024. 11937559 10.4049/jimmunol.168.8.4018

[21] Hsu A, Aronoff DM, Phipps J, Goel D, Mancuso P (2007) Leptin improves pulmonary bacterial clearance and survival in ob/ob mice during pneumococcal pneumonia. Clin Exp Immunol 150: 332–339. 17822444 10.1111/j.1365-2249.2007.03491.x PMC2219341

[22] Moore SI, Huffnagle GB, Chen GH, White ES, Mancuso P (2003) Leptin modulates neutrophil phagocytosis of Klebsiella pneumoniae. Infect Immun 71: 4182–4185. 12819114 10.1128/IAI.71.7.4182-4185.2003 PMC161963

[23] Ikejima S, Sasaki S, Sashinami H, Mori F, Ogawa Y, et al (2005) Impairment of host resistance to Listeria monocytogenes infection in liver of db/db and ob/ob mice. Diabetes 54: 182–189. 15616027 10.2337/diabetes.54.1.182

[24] Madan R, Guo X, Naylor C, Buonomo EL, Mackay D, et al (2014) Role of Leptin-Mediated Colonic Inflammation in Defense against Clostridium difficile Colitis. Infect Immun 82: 341–349. 24166957 10.1128/IAI.00972-13 PMC3911837

[25] Ordway D, Henao-Tamayo M, Smith E, Shanley C, Harton M, et al (2008) Animal model of Mycobacterium abscessus lung infection. J Leukoc Biol 83: 1502–1511. 18310351 10.1189/jlb.1007696

[26] Wieland CW, Florquin S, Chan ED, Leemans JC, Weijer S, et al (2005) Pulmonary Mycobacterium tuberculosis infection in leptin-deficient ob/ob mice. Int Immunol 17: 1399–1408. 16141243 10.1093/intimm/dxh317

[27] Guo X, Roberts MR, Becker SM, Podd B, Zhang Y, et al (2011) Leptin signaling in intestinal epithelium mediates resistance to enteric infection by Entamoeba histolytica. Mucosal Immunol 4: 294–303. 21124310 10.1038/mi.2010.76 PMC3079783

[28] Fernandez-Riejos P, Najib S, Santos-Alvarez J, Martin-Romero C, Perez-Perez A, et al (2010) Role of leptin in the activation of immune cells. Mediators Inflamm 2010: 568343. 20368778 10.1155/2010/568343 PMC2846344

[29] Mancuso P, Huffnagle GB, Olszewski MA, Phipps J, Peters-Golden M (2006) Leptin corrects host defense defects after acute starvation in murine pneumococcal pneumonia. Am J Respir Crit Care Med 173: 212–218. 16210671 10.1164/rccm.200506-909OC

[30] Bartlett JG (2011) Diagnostic tests for agents of community-acquired pneumonia. Clin Infect Dis 52 Suppl 4S296–304. 21460288 10.1093/cid/cir045

[31] Mancuso P, Peters-Golden M, Goel D, Goldberg J, Brock TG, et al (2011) Disruption of leptin receptor-STAT3 signaling enhances leukotriene production and pulmonary host defense against pneumococcal pneumonia. J Immunol 186: 1081–1090. 21148797 10.4049/jimmunol.1001470 PMC3133444

[32] Phipps JC, Aronoff DM, Curtis JL, Goel D, O′Brien E, et al (2010) Cigarette smoke exposure impairs pulmonary bacterial clearance and alveolar macrophage complement-mediated phagocytosis of Streptococcus pneumoniae. Infect Immun 78: 1214–1220. 20008540 10.1128/IAI.00963-09 PMC2825918

[33] Naggert JK, Fricker LD, Varlamov O, Nishina PM, Rouille Y, et al (1995) Hyperproinsulinaemia in obese fat/fat mice associated with a carboxypeptidase E mutation which reduces enzyme activity. Nat Genet 10: 135–142. 7663508 10.1038/ng0695-135

[34] Cawley NX, Zhou J, Hill JM, Abebe D, Romboz S, et al (2004) The carboxypeptidase E knockout mouse exhibits endocrinological and behavioral deficits. Endocrinology 145: 5807–5819. 15358678 10.1210/en.2004-0847

[35] Johnston RA, Theman TA, Shore SA (2006) Augmented responses to ozone in obese carboxypeptidase E-deficient mice. Am J Physiol Regul Integr Comp Physiol 290: R126–133. 16002559 10.1152/ajpregu.00306.2005

[36] Xu H, Barnes GT, Yang Q, Tan G, Yang D, et al (2003) Chronic inflammation in fat plays a crucial role in the development of obesity-related insulin resistance. J Clin Invest 112: 1821–1830. 14679177 10.1172/JCI19451 PMC296998

[37] Herishanu Y, Rogowski O, Polliack A, Marilus R (2006) Leukocytosis in obese individuals: possible link in patients with unexplained persistent neutrophilia. Eur J Haematol 76: 516–520. 16696775 10.1111/j.1600-0609.2006.00658.x

[38] Morgan OW, Bramley A, Fowlkes A, Freedman DS, Taylor TH, et al (2010) Morbid obesity as a risk factor for hospitalization and death due to 2009 pandemic influenza A(H1N1) disease. PLoS One 5: e9694. 20300571 10.1371/journal.pone.0009694 PMC2837749

[39] Paich HA, Sheridan PA, Handy J, Karlsson EA, Schultz-Cherry S, et al (2013) Overweight and obese adult humans have a defective cellular immune response to pandemic H1N1 Influenza a virus. Obesity(Silver Spring) 10.1002/oby.20383 PMC3695020 23512822

[40] Cawley NX, Wetsel WC, Murthy SR, Park JJ, Pacak K, et al (2012) New roles of carboxypeptidase E in endocrine and neural function and cancer. Endocr Rev 33: 216–253. 22402194 10.1210/er.2011-1039 PMC3365851

[41] Surwit RS (1988) Diet-induced type II diabetes in C57BL/6J mice. Diabetes (New York, NY) 37: 1163–1167. 10.2337/diab.37.9.1163 3044882

[42] Hariri N, Thibault L (2010) High-fat diet-induced obesity in animal models. Nutrition Research Reviews 23: 270–299. 20977819 10.1017/S0954422410000168

[43] Strandberg L, Verdrengh M, Enge M, Andersson N, Amu S, et al (2009) Mice Chronically Fed High-Fat Diet Have Increased Mortality and Disturbed Immune Response in Sepsis. PLoS ONE 4: e7605. 19865485 10.1371/journal.pone.0007605 PMC2765728

[44] Seminog OO, Goldacre MJ (2013) Risk of pneumonia and pneumococcal disease in people with severe mental illness: English record linkage studies. Thorax 68: 171–176. 23242947 10.1136/thoraxjnl-2012-202480

[45] Rueda AM, Ormond M, Gore M, Matloobi M, Giordano TP, et al (2010) Hyperglycemia in diabetics and non-diabetics: effect on the risk for and severity of pneumococcal pneumonia. J Infect 60: 99–105. 20005251 10.1016/j.jinf.2009.12.003

[46] Rivas-Santiago B, Trujillo V, Montoya A, Gonzalez-Curiel I, Castaneda-Delgado J, et al (2012) Expression of antimicrobial peptides in diabetic foot ulcer. J Dermatol Sci 65: 19–26. 22047630 10.1016/j.jdermsci.2011.09.013

[47] Cianfarani F, Toietta G, Di Rocco G, Cesareo E, Zambruno G, et al (2013) Diabetes impairs adipose tissue-derived stem cell function and efficiency in promoting wound healing. Wound Repair Regen 21: 545–553. 23627689 10.1111/wrr.12051

[48] Louriz M, Mahraoui C, Azzouzi A, El Fassy Fihri MT, Zeggwagh AA, et al (2010) Clinical features of the initial cases of 2009 pandemic influenza A (H1N1) virus infection in an university hospital of Morocco. Int Arch Med 3: 26. 20979619 10.1186/1755-7682-3-26 PMC2987860

[49] Nanji AA, Freeman JB (1985) Relationship between body weight and total leukocyte count in morbid obesity. Am J Clin Pathol 84: 346–347. 4036865 10.1093/ajcp/84.3.346

[50] Hodgson KA, Govan BL, Walduck AK, Ketheesan N, Morris JL (2013) Impaired Early Cytokine Responses at the Site of Infection in a Murine Model of Type 2 Diabetes and Melioidosis Comorbidity. Infection and Immunity 81: 470–477. 23208607 10.1128/IAI.00930-12 PMC3553796

[51] Faggioni R, Jones-Carson J, Reed DA, Dinarello CA, Feingold KR, et al (2000) Leptin-deficient (*ob/ob*) mice are protected from T cell-mediated hepatotoxicity: Role of tumor necrosis factor alpha and IL-18. PNAS 97: 2367–2372. 10681432 10.1073/pnas.040561297 PMC15807

[52] Tilg H, Moschen AR (2008) Role of adiponectin and PBEF/visfatin as regulators of inflammation: involvement in obesity-associated diseases. Clin Sci (Lond) 114: 275–288. 18194136 10.1042/CS20070196

